# Factors affecting the decision to investigate older adults with potential cancer symptoms: a systematic review

**DOI:** 10.3399/BJGP.2021.0257

**Published:** 2021-11-16

**Authors:** Daniel Jones, Erica Di Martino, Stephen H Bradley, Blessing Essang, Scott Hemphill, Judy M Wright, Cristina Renzi, Claire Surr, Andrew Clegg, Niek De Wit, Richard Neal

**Affiliations:** Leeds Institute of Health Sciences, University of Leeds, Leeds, UK.; Leeds Institute of Health Sciences, University of Leeds, Leeds, UK.; Leeds Institute of Health Sciences, University of Leeds, Leeds, UK.; Leeds Institute of Health Sciences, University of Leeds, Leeds, UK.; Leeds Institute of Health Sciences, University of Leeds, Leeds, UK.; Leeds Institute of Health Sciences, University of Leeds, Leeds, UK.; University College London, London, UK.; Leeds Beckett University, Leeds, UK.; Academic Unit for Ageing & Stroke Research, University of Leeds, Leeds, UK.; Julius Center for Health Sciences and Primary Care, University Medical Center Utrecht, Utrecht, the Netherlands.; School of Medicine, University of Leeds, Leeds, UK.

**Keywords:** cancer, decision making, shared, early detection of cancer, frail elderly, primary health care, systematic review

## Abstract

**Background:**

Older age and frailty increase the risk of morbidity and mortality from cancer surgery and intolerance of chemotherapy and radiotherapy. The effect of old age on diagnostic intervals is unknown; however, older adults need a balanced approach to the diagnosis and management of cancer symptoms, considering the benefits of early diagnosis, patient preferences, and the likely prognosis of a cancer.

**Aim:**

To examine the association between older age and diagnostic processes for cancer, and the specific factors that affect diagnosis.

**Design and setting:**

A systematic literature review.

**Method:**

Electronic databases were searched for studies of patients aged >65 years presenting with cancer symptoms to primary care considering diagnostic decisions. Studies were analysed using thematic synthesis and according to the Synthesis Without Meta-analysis guidelines.

**Results:**

Data from 54 studies with 230 729 participants were included. The majority of studies suggested an association between increasing age and prolonged diagnostic interval or deferral of a decision to investigate cancer symptoms. Thematic synthesis highlighted three important factors that resulted in uncertainty in decisions involving older adults: presence of frailty, comorbidities, and cognitive impairment. Data suggested patients wished to be involved in decision making, but the presence of cognitive impairment and the need for additional time within a consultation were significant barriers.

**Conclusion:**

This systematic review has highlighted uncertainty in the management of older adults with cancer symptoms. Patients and their family wished to be involved in these decisions. Given the uncertainty regarding optimum management of this group of patients, a shared decision-making approach is important.

## INTRODUCTION

Worldwide the population of those aged >65 years is growing faster than any other age group.^1^ The burden of cancer falls predominantly on older patients, with half of all new diagnoses occurring in people aged >70 years and incidence rates for all cancers increasing most rapidly in the >75 years age group.^1^^,^^2^ The benefits of asymptomatic cancer screening in older adults are unproven and, in most countries, it is not recommended.^3^^,^^4^ In countries such as the UK, symptomatic presentation to primary care is the most frequent route to a cancer diagnosis in older adults.^5^

Diagnosing cancer at an early stage is important, and associated with improved survival.^6^ In older adults, these survival benefits are likely to be reduced because of shorter life expectancy. If cancer is diagnosed, older patients who are frail have an increased risk of morbidity and mortality from cancer surgery, and intolerance to chemotherapy and radiotherapy.^7^ As a result, the management of older adults with cancer symptoms in primary care is difficult.^8^^,^^9^ Older adults need a balanced approach to the diagnosis and management of cancer symptoms. The imperative to diagnose cancer early in older adults must be balanced against the prognosis of the cancer, the likely success and tolerance of treatment, the presence of comorbidities, and patient preferences. Some older adults favour quality rather than length of life,^10^ are less likely to want investigation for cancer symptoms, and would accept a higher risk of cancer being undiagnosed.^11^

The aim of this review is to consider the global literature on the association between old age and the diagnostic process for cancer. The objectives were:

- to explore the effect of increasing age on the primary care interval (the time from first presentation to referral) in the diagnosis of cancer;
- to identify the factors that influence the decision to investigate potential cancer symptoms in older adults in primary care, both from a patient and healthcare professional perspective; and
- to understand how the factors identified have an impact on decision making, processes, and outcomes.

## METHOD

### Protocol

Prior to commencing this review, the study protocol was registered with PROSPERO (reference number: CRD42020180656). The review has been conducted and reported in accordance with the *Cochrane Handbook for Systematic Reviews of Interventions*^12^ and the PRISMA statement.^13^

**Table:** How this fits in

There is uncertainty in the management of cancer symptoms in primary care. This is the first review, to the authors’ knowledge, to consider the effect of older age on decision making by patients and GPs when patients present to primary care with cancer symptoms. Multiple factors were found to influence the patient and GP decision to investigate cancer symptoms including the presence of frailty, comorbidities, and cognitive impairment; family and carer involvement; and consultation time. Given the uncertainty, a shared decision-making approach is appropriate, but in routine general practice this may be difficult to achieve, mostly because of a lack of time within the consultation.

### Definition of older adults

There is no universally accepted age threshold for defining old age. The World Health Organization’s definition of ‘older people’ as those aged ≥65 years was adopted in this study.^14^

### Eligibility criteria

Any studies (qualitative and quantitative) of patients aged ≥65 years or with a subgroup of patients aged ≥65 years with symptoms and signs that warrant investigation and referral for suspected cancer presenting to primary care before diagnosis were included. Case–control, cohort, and cross- sectional studies were included as well as interview and focus group studies. Editorials, single/clinical case studies, reviews, expert opinion articles, and studies that were published as abstracts were excluded from the review.

### Search strategy

On 29 April 2020 electronic databases (Box 1) were searched for published and unpublished studies of cancer-related shared decision making (SDM) for older adults in primary care. See Supplementary Appendix S1 for full search strategies.

**Box 1.** Systematic review search strategy

Applied Social Sciences Index and Abstracts (ASSIA) (ProQuest) 1987 to present CINAHL (EBSCOhost) 1981 to present Cochrane Central Register of Controlled Trials (Wiley): Issue 4 of 12, April 2020 Cochrane Database of Systematic Reviews (Wiley): Issue 4 of 12, April 2020 EMBASE Classic+EMBASE (Ovid) 1947 to 27 April 2020 Ovid MEDLINE(R) and Epub Ahead of Print, In-Process & Other Non-Indexed Citations and Daily 1946 to 28 April 2020 APA PsycINFO (Ovid) 1806 to April Week 3 2020 Web of Science Core Collection: Citation Indexes (Clarivate Analytics) 1900 to present ISRCTN registry (Springer) ClinicalTrials.gov (US National Institutes of Health) Evidence Search (National Institute for Health and Care Excellence)

Subject headings and free-text words were identified for use in the search concepts by the study authors and based on the search strategy published in a similar review.^15^ No limits (for example, language or date of publication) were applied to the search. The searches were peer reviewed by a second information specialist.

Further relevant studies were sought by searching the citations of included studies, and hand searches of conference abstracts (Cancer and Primary Care Research International Network, National Cancer Research Institute, Macmillan Cancer Support, and Cancer Research UK).

### Data collection

All titles and abstracts were independently reviewed by two authors. Any disagreements were resolved through discussion or through adjudication by a third author. Reasons for exclusion were recorded. Data extraction was undertaken using a data extraction template.

### Risk of bias of included studies

The mixed-methods appraisal tool (MMAT) was used to assess the risk of bias for the included studies.^16^ The reviewers’ reasons for ratings, including strengths and weaknesses of studies, were recorded independently by two authors before agreeing on a final score.

### Synthesis of results

Meta-analysis was not possible because of the heterogeneity of the included studies. Quantitative studies were therefore analysed using the SWiM (Synthesis Without Meta-analysis) reporting guidelines and checklist.^17^ Qualitative studies were analysed using thematic synthesis described by Thomas and Harden.^18^ Quotes and supporting information were extracted using a template and imported into NVivo (version 12). Quotes and text were then coded line by line before the development of descriptive and analytic themes that enabled comparisons and synthesis between studies. This synthesis was undertaken independently by two reviewers. The ENTREQ guidelines were followed for reporting the synthesis of qualitative research.^19^ Following the separate analysis of qualitative and quantitative data, the findings were combined by considering the barriers and facilitators to decision making in primary care. This method was based on previous published guidance on integrating qualitative research in systematic reviews.^20^

## RESULTS

**Figure 1.**
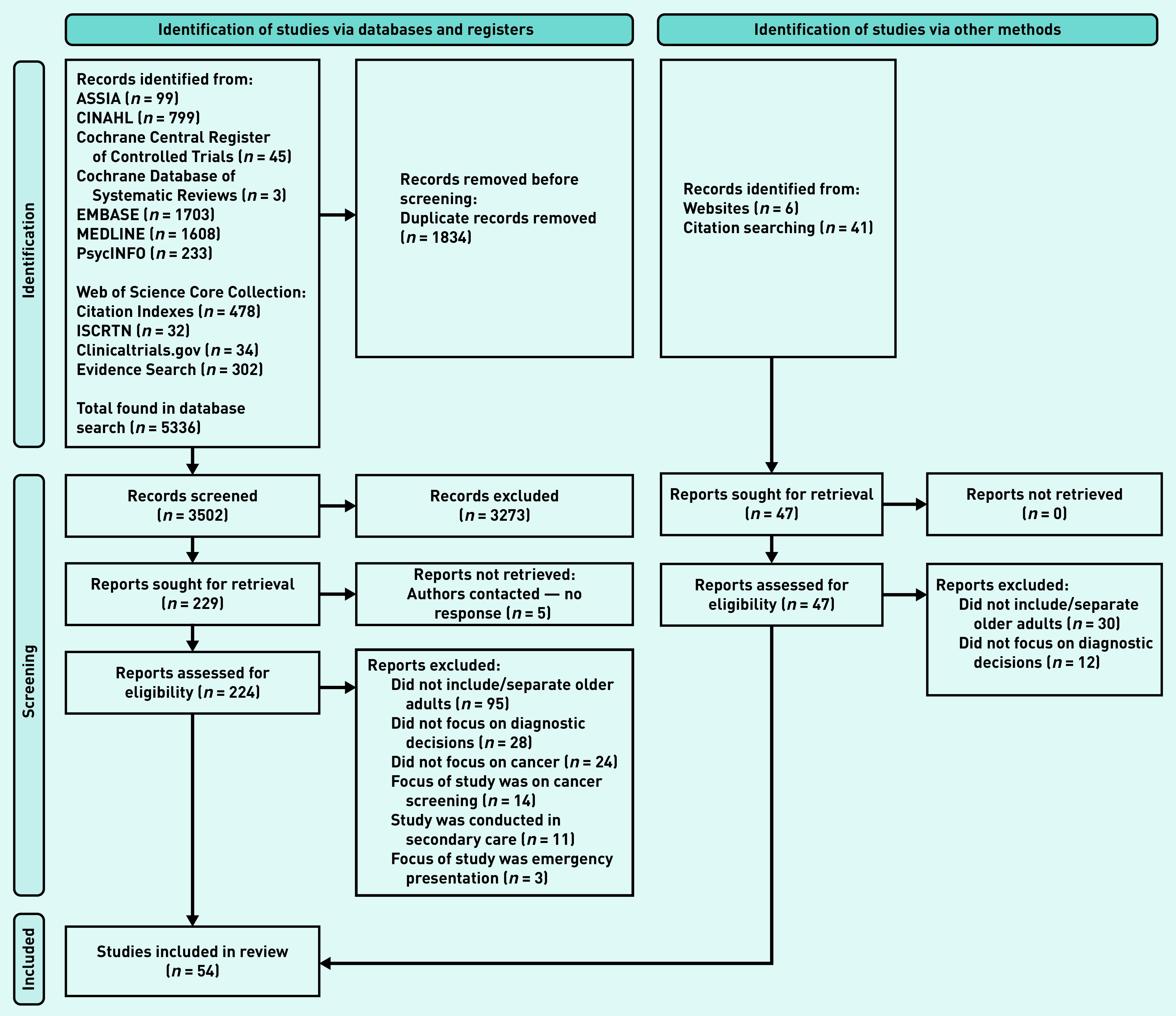
*PRISMA flow diagram.*

The database searches identified 5336 studies. After title and abstract screening and full-text review, 54 articles were included with 230 729 participants (Figure 1). Studies ranged in size from 9 to 109 433 participants. In total, 29 articles included quantitative data,^11^^,^^21^^–^^48^ 24 provided qualitative data,^49^^–^^72^ and one included both qualitative and quantitative data.^48^ A variety of study settings and cancer types were included (see Supplementary Tables S1 and S2). Overall, the quality of studies was judged to be high with an average MMAT across the 54 included studies of 4.6/5.

### Quantitative study results

The 29 quantitative studies included in this review are summarised in Supplementary Table S1. A variety of cancers were investigated in a number of different countries. The association between increasing age and the investigation and referral of cancer symptoms was not related to the type of cancer being investigated or the study setting. Outcomes considered by the studies included the association between increasing age and decision making on cancer investigations and referral, and on the primary care interval as defined by the Aarhus Statement.^49^

Fifteen studies considered the length of the primary care interval. Seven studies reported that age was not associated with time to referral or diagnosis,^28^^–^^34^ five reported that increasing age was associated with a prolonged diagnostic interval,^23^^–^^27^ and three reported increasing age resulted in shorter diagnostic intervals.^21^^,^^22^^,^^48^

Eleven studies considered the association between increasing age and GP factors that may affect the decision to investigate cancer symptoms. These factors included suspicion of cancer, cancer referral, anticipated regret (because of missed diagnosis), and loss of continuity of care. Two studies found that increasing age was associated with GP factors that would prompt a decision to investigate cancer symptoms.^35^^,^^36^ However, five studies suggested that increasing age was associated with factors that would prevent or delay the investigation of cancer symptoms.^27^^,^^37^^–^^40^ The remaining four studies found that increasing age was not associated with GP factors on decisions to investigate or refer cancer symptoms.^32^^,^^41^^,^^42^^,^^47^

Five studies considered the association between increasing age and patient aspects of the diagnostic process. Two studies found that, with increasing age, patient factors such as declining investigations and not attending appointments were more common.^11^^,^^43^ Three studies found that age was not associated with patients’ preference to proceed with investigations for suspected prostate cancer, patients’ wish for cancer investigations, or attitudes towards a cancer diagnosis.^44^^–^^46^

**Figure 2.**
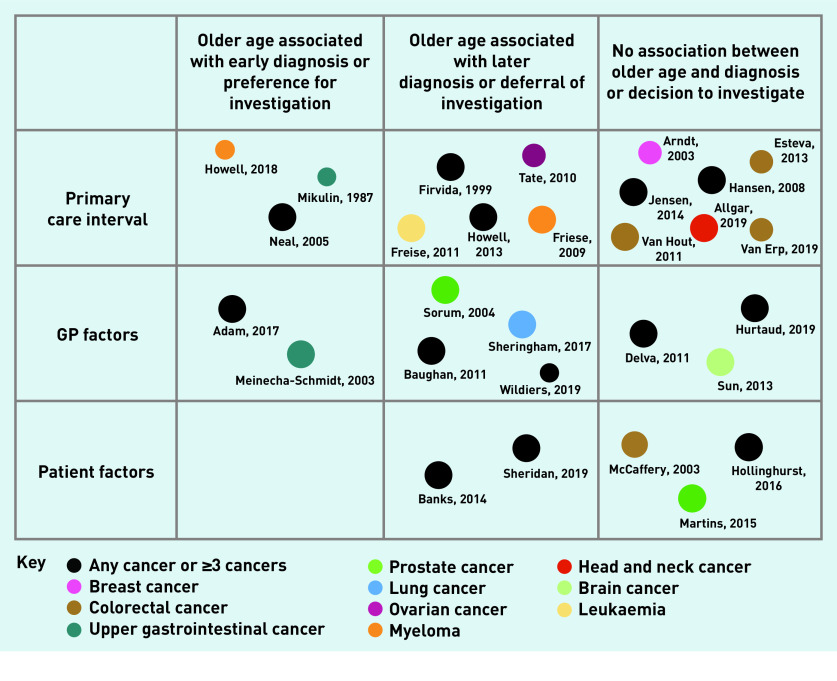
*Diagram to show the number of quantitative studies, the association with cancer diagnosis, the cancer investigated, and the quality assessment. Size of circle corresponds to the quality rating judged using MMAT.* *MMAT = mixed-methods appraisal tool.*

These results of the quantitative analysis are summarised in Figure 2. The qualitative results below go some way to explaining these findings.

### Qualitative study findings

Twenty-five studies included in the review provided qualitative data on the association with age and the primary care interval (Supplementary Table S2).^48^^,^^50^^–^^73^ Thematic synthesis identified the following themes on the decision to investigate or refer cancer symptoms in older adults: the effect of old age on GP and patient decision making; frailty, cognitive impairment, and comorbidities; involving family and carers in decision making; and consultation time. The difficulty of providing adequate time within the primary care consultation for older adults was also highlighted.

#### The effect of old age on GP and patient decision making

The included studies suggested the presence of significant variation in how GPs and patients managed cancer symptoms in an ageing population. There was evidence that older adults faced additional barriers to diagnosis, with cancer symptoms sometimes being attributed to the ‘effects of old age’ by both patients and GPs. Data suggested that GPs could make decisions on behalf of patients but this may result in depersonalisation and a loss of autonomy. One study suggested that GPs may apply their own personal values to decision making, which could be at odds with that of the patient.^69^ In contrast, there was also evidence of doctors considering quality of life and life expectancy when making decisions rather than age alone, which affected the likelihood of investigation and referral. These findings are summarised in Box 2.

**Box 2.** Themes and illustrative quotes demonstrating the effect of old age on GP and patient decision making

**Theme**	**Subtheme**	**Illustrative quotes**
Old age alone can affect decision making	Possible cancer symptoms are attributed to the ageing process	A GP stated:
		*‘I find that there can be a short delay in management, because we tend to trivialise symptoms that can be attributed to age, fatigue, asthenia, maybe a slight anaemia, things that are relatively trivial. We tend to say it’s just age.’* ^56^
		A patient stated:
		*‘It was … a gradual process, which I put down to old age …* [and] *I’d had a bad back, so I was quite sort of willing to accept that my back hurts a bit.’* ^48^
	Healthcare professionals may make decisions on behalf of older patients	*‘With an elderly patient, certain specialists and general practitioners have a tendency to make the decision on behalf of the patient, which is an important problem.’* ^56^
	Healthcare professionals’ personal values may be at odds with those of the patient and carers	The carer recalled that the GP stated:
		*‘… for what life shall we save him?’*
		The carer further reported:
		*‘… it was like he didn’t want anything to be done, that there was no point in doing anything, and that we should be satisfied with taking the world as we found it.’* ^69^
	Old age alone should not delay investigation and referral	A GP stated:
		*‘I have folks* [in whom] *we’re doing certain tests and things well beyond what generally is recommended but I think for good reason … I have a couple patients in their late 80s and 90s where I tell them: “you’re likely to live another decade or two … so we might need to be a little more aggressive … ”.’* ^71^

#### Frailty, cognitive impairment, and comorbidities

Frailty, comorbidities, and cognitive impairment were highlighted as important themes throughout the qualitative synthesis, and are summarised in Box 3. The study analysis suggested that GPs undertook an assessment of a patient’s overall health or frailty when making decisions about the investigation or referral for cancer symptoms. Older adults deemed to be frail or in poor health were less likely to be investigated or referred if they developed cancer symptoms. However, the evidence base behind these assessments of frailty were questioned by patients and GPs. There was a concern that GPs could overestimate frailty, especially with older adults in care homes, which could negatively affect the investigation and referral for cancer symptoms in these patients.

**Box 3.** Themes and illustrative quotes demonstrating the effect of frailty, cognitive impairment, and comorbidities

**Theme**	**Subtheme**	**Illustrative quotes**
Frailty	Healthcare professionals are informally assessing frailty	A GP stated:
		*‘I’ve never used any specific scale to assess anyone’s frailty. I don’t know what the evidence is behind that frailty score.’* ^50^
	Healthcare professionals are less likely to investigate or refer patients they deem to be frail	A GP stated:
		*‘We need to consider … the psychological and organic weaknesses that mean, possibly justifiably, that we shouldn’t do as much as we would with a younger person.’* ^56^
		A GP stated:
		*‘This woman in her 80s had a breast mass … she* [had] *poor life expectancy, she was already on home oxygen, heart failure, all those comorbid conditions, we could see her lungs failing, and I don’t think we need to do anything about* [the breast mass] *.’*^71^
	Assumptions about frailty may prevent investigation	A GP stated:
		*‘There may be a degree* [of] *assumption going on “I don’t think Mrs Bloggs is well enough” and I wonder whether there is a better way …’* ^50^

Cognitive impairment	Healthcare professionals are less likely to investigate or refer older patients they deem to be cognitively impaired	A GP stated:
		*‘Well, I don’t push the investigation or anything … For me, it’s really a complete hindrance to send people for investigations to seek out cancerous pathology.’* ^56^
	Older patients with cognitive impairment may be distressed by examinations or investigations	An older patient with dementia who had undergone tests for colorectal cancer stated:
		*‘That woman who ran around and hurt me. Well, she didn’t know what she were doing. “No!” I kept saying to her. I said “It’s not right!” Two people hit at me.’*
		The study reported that there were also signs of distress during the interview, *’contorting her face’* and *‘wringing her hands with worry’*, which showed the pain and distress of undergoing intimate clinical investigations.^63^
	The presence of cognitive impairment can affect communication with healthcare professionals	A 79-year-old with colorectal cancer stated it was his wife who had noticed the patient’s symptoms:
		*‘I have Alzheimer’s disease and my wife noticed the change in bowel habits. I had no other signs or symptoms.’* ^67^
	Despite cognitive impairment, patients may be fit and investigation could be warranted	A GP stated:
		*‘Even if they’re very cognitively impaired, we can still share plenty of things, and often they find that it’s worthwhile to continue to fight.’* ^56^

Comorbidities	Investigation and referral of symptoms possibly owing to cancer were delayed because of comorbidities	The carer of a 78-year-old man recalled how the GP attributed his signs of illness to pain from a knee replacement:
		*‘We said, you know, he’s really finding it hard to mobilise and you know, loss of appetite and depression. And,* [the GP] *instead of looking for another reason, it was, “oh well, he’s in pain. You know, if you sort the pain out, we’ll sort the other bits out”.’* ^55^
		A 72-year-old woman with ovarian cancer and longstanding back pain reported:
		*‘I first visited my doctor about my symptoms … I was not examined. I was told the pain was coming from my back (I had a back problem for years).’* ^67^
		*‘I’ve got COPD* [chronic obstructive pulmonary disease] *but I never coughed up blood before. I thought it will clear up but after two weeks it didn’t so I thought I had better get it checked.’* ^53^
	Annual check-ups for comorbidities resulted in opportunities for earlier diagnosis	*‘The cancer was only found on annual chest check for COPD.’* ^67^
		A patient (aged between 85 and 89 years) with lung cancer stated:
		*‘I go six monthly to the nurse in the clinic and I mentioned to her I was spitting blood and she said “well make an appointment with the doctor” …’* ^53^

The presence of cognitive impairment had a similar effect to that of frailty in the study analysis. Some GPs were less likely to investigate patients with dementia because of the perception that patients with cognitive impairment may be distressed by medical examinations or investigations and not benefit from a diagnosis of cancer. However, it was also recognised that patients with cognitive impairment may be physically fit and have a good quality of life, and as a result that it was necessary to ‘continue to fight’, as one GP put it.^56^ The presence of cognitive impairment was also identified as a barrier to SDM.

The study analysis suggest that comorbidities such as osteoarthritis, chronic back pain, chronic obstructive pulmonary disease, cerebrovascular disease, and anxiety and depression were attributed as a cause for symptoms by both GPs and patients. This frequently resulted in a delay in the investigation or referral for cancer symptoms. However, there were also examples of cancer symptoms being investigated as a result of attending routine health checks for comorbidities.

#### Involving family and carers in decision making

As a result of advanced age, frailty, or cognitive impairment, there were frequent discussions about the impact of a patient’s family or carers on the decisions made in primary care, especially in patients with cognitive impairment (Box 4).

**Box 4.** Themes and illustrative quotes demonstrating the impact of a patient’s family or carers on the decisions made in primary care

**Theme**	**Subtheme**	**Illustrative quotes**
Family and carers	Family and carers should be involved in decision making in patients with cognitive impairment	A GP stated:
		*‘If we’re referring to patients with advanced cognitive impairment … it’s obvious that the decision should be taken with the carers, those close to the patient, their family …’* ^56^
	The wishes of family and carers may be at odds with the wishes of the patient	A GP stated:
		*‘A 50-year-old who says to you “If I’m ever in that position, let me go, don’t insist, let me die or help me to die”, but when they* [the family] *face that situation* [later in life]*, if the smallest door of hope opens, they* [the family] *take it; it’s normal.’* ^56^
	What level of responsibility should be taken by the family or carers?	*‘Uncertainty extended to knowing how much, or how little, they* [the family] *were to be involved in the clinical investigations consent process of their relative with dementia and what level of responsibility — if any — they shouldered in taking such a decision.’* ^63^

The studies suggested that, if patients had advanced cognitive impairment, then that patient’s family and carers should be involved in decisions on investigation of cancer symptoms. However, there was concern that the wishes of the family and carers may not support those of the patient. There were also concern over the level of responsibility that should be managed by relatives.

#### Consultation time

It was recognised by both patients and GPs that time constraints within the consultation could limit the communication of symptoms by patients. Although a lack of time in the consultation could affect patients of all ages, it was more likely to affect older adults because of the presence of frailty, comorbidities, and cognitive impairment, resulting in more complex consultations.

A study of GPs found that most were aware that time constraints within consultations with older adults limited what could be discussed.^60^ Two studies highlighted problems with policies such as ‘one appointment, one problem’, which may not suit an older patient demographic.^74^^,^^75^ However, there was evidence that GPs considered the practicalities of older adults attending appointments, with one GP suggesting that older adults may find afternoon appointments easier.^60^

## DISCUSSION

### Summary

To the authors’ knowledge, this is the first systematic review to explore the effect of old age on the investigation and referral of cancer in primary care. The majority of studies suggest a possible association between increased age and a prolonged diagnostic interval or deferred cancer investigations. The findings suggest that, for patients and GPs, deciding how to manage older patients with symptoms that could herald a cancer diagnosis is challenging. As well as an assessment of the patient’s wishes, such decisions often require an assessment of patients’ overall health or frailty, along with a judgement as to whether the harms of investigation or referral would be justified by benefits.

There is significant variation in the findings of the studies included in this review. Some studies found that older adults and those with high levels of frailty or comorbidity had prolonged diagnostic intervals or were not investigated for possible cancer, which were in direct contrast with other studies in the same patient group. This variation may reflect uncertainty and a lack of evidence regarding the management of cancer symptoms in older adults. Judgements undertaken by GPs based on a patient’s age or perceived frailty could result in inconsistency and a high degree of variation in clinical practice. However, the variation could also be the result of well-balanced decisions to postpone investigations because of a low likelihood of benefit from a cancer diagnosis and a higher risk of complications from cancer investigations or treatment. Finally, it may be a consequence of patient preference in shared decisions around investigation.

The review has highlighted both patients’ and their families’ wishes to be involved in decisions around care. Given the uncertainty regarding optimum management of this group of patients, an SDM approach is likely to be helpful. However, it is not clear how best to implement this, and several barriers to its use were highlighted in this review, most notably the presence of cognitive impairment and the need for additional time within a consultation to fully inform the patient and allow for SDM.

### Strengths and limitations

This large systematic review of 54 studies has been robustly carried out and demonstrates important and novel findings for patients and primary care practitioners. Studies were included from a variety of countries and investigated a wide range of cancer types. The heterogeneity of included studies precluded meta-analysis and may have also resulted in contrasting findings. There is inconsistency in the use of terms to describe the primary care interval within the literature and included studies. This was addressed in the current study by reviewing the details of each study against the inclusion criteria. The MMAT for quality assessment was chosen as both qualitative and quantitative studies were included; however, the tool was found to be limited in discriminating study quality. Finally, many of the included studies, particularly the qualitative ones, included few participants and as a result may not be generalisable to the older adult population as a whole.

### Comparison with existing literature

Although this is the first review, to the authors’ knowledge, to consider the effect of age on diagnostic decisions, there are multiple studies concerning the treatment decisions of older adults who are frail and have a diagnosis of cancer. These studies are largely based in secondary care and have other competing factors to consider, such as the side effects of potential treatments and the chances of success. However, the effect of age, frailty, comorbidities, and cognitive impairment are frequently highlighted in these studies. Overall, the results of this review are supported by the findings of work undertaken on cancer treatment.

A systematic review on the effect of frailty on cancer outcomes found that patients with cancer and a diagnosis of frailty had increased all-cause mortality, increased postoperative mortality, and more frequent complications of treatment than patients with cancer without a diagnosis of frailty.^7^ A review on the impact of comorbidity on cancer treatment found similar results. The review reports that patients with comorbidity had poorer survival, poorer quality of life, and higher healthcare costs than those without comorbidities.^76^ A systematic review on the effect of dementia on cancer outcomes found that patients with dementia and cancer had a reduced likelihood of receiving: cancer screening, cancer staging information, cancer treatment with curative intent, and pain management compared with those with cancer only.^77^ A qualitative study on the information needs of patients with dementia making decisions about cancer treatment found that cancer treatment was adjusted because of dementia; that there were difficulties in communicating clinical information that resulted in the frequent involvement of informal caregivers; and a need for information on the functional impact of dementia and how this will affect cancer treatment.^78^ These studies are largely based in secondary care and have other competing factors to consider, such as the side effects of potential treatments and the chances of success. However, the effect of age, frailty, comorbidities, and cognitive impairment are frequently highlighted in these studies. Overall, the results of this review are supported by the findings of work undertaken on cancer treatment.

### Implications for research and practice

National guidelines on investigation and referral of patients with cancer symptoms do not consider older age or frailty.^1^^,^^79^ However, the question of whether healthcare professionals should treat older adults with cancer symptoms differently remains.^9^ It is not possible to make appropriate management decisions on the basis of age alone, as many patients remain active and healthy well into advanced age, or may express preferences about investigation and treatment. Even patients who may not be able to tolerate aggressive cancer treatments might still benefit from diagnosis, for example, should they wish to know about prognosis or to access palliative care. This review highlights uncertainty in both patients’ and GPs’ views and decisions surrounding the investigation and referral of older adults with cancer symptoms. In this context of uncertainty, an SDM approach is most appropriate.^9^ This would allow patients, and in some cases their family, to evaluate the pros and cons of diagnostic referral on an individual basis. SDM is a key part of the NHS Long Term Plan,^80^ which advocates personalised care across the whole care system.

Barriers to the use of SDM, however, were apparent in the review. The authors of this current study consider in a primary care consultation that there is insufficient time to fully undertake SDM, with the presence of cognitive impairment, comorbidities, and frailty; complex medical/social circumstances; the need for assessments of capacity; and to involve family members. Significant work has been undertaken to understand the use of SDM and holistic geriatric assessment tools to aid decision making for cancer treatment.^81^ These barriers might be addressed by further use of pre-diagnostic frailty scoring systems and holistic assessments of older adults, and may benefit from further development of geriatric oncology services expanding into primary care, as has been suggested in work considering frailty and cancer treatment.^7^

## Data

The full search strategy and all data are available in the supplementary material.
